# Prior Activation of 5-HT7 Receptors Modulates the Conditioned Place Preference With Methylphenidate

**DOI:** 10.3389/fnbeh.2019.00208

**Published:** 2019-09-18

**Authors:** Cristiana Carbone, Sara Lucia Maria Lo Russo, Enza Lacivita, Annika Frank, Enrico Alleva, Holger Stark, Luciano Saso, Marcello Leopoldo, Walter Adriani

**Affiliations:** ^1^Center for Behavioral Sciences and Mental Health, Istituto Superiore di Sanità, Rome, Italy; ^2^Dipartimento di Farmacia—Scienze del Farmaco, Università degli Studi di Bari, Bari, Italy; ^3^Institute of Pharmaceutical and Medicinal Chemistry, Heinrich Heine University Düsseldorf, Düsseldorf, Germany; ^4^Department of Physiology and Pharmacology “V. Erspamer”, Sapienza University, Rome, Italy; ^5^BIOFORDRUG s.r.l., Università degli Studi di Bari, Bari, Italy

**Keywords:** reward processing, behavioral adaptation, context evaluation, 5-HT, DA, memory consolidation, synaptic plasticity

## Abstract

The serotonin receptor subtype 7 (5-HT7R) is clearly involved in behavioral functions such as learning/memory, mood regulation and circadian rhythm. Recent discoveries proposed modulatory physiological roles for serotonergic systems in reward-guided behavior. However, the interplay between serotonin (5-HT) and dopamine (DA) in reward-related behavioral adaptations needs to be further assessed. TP-22 is a recently developed arylpiperazine-based 5-HT7R agonist, which is also showing high affinity and selectivity towards D1 receptors. Here, we report that TP-22 displays D1 receptor antagonist activity. Moreover, we describe the first *in vivo* tests with TP-22: first, a pilot experiment (assessing dosage and timing of action) identified the 0.25 mg/kg i.v. dosage for locomotor stimulation of rats. Then, a conditioned place preference (CPP) test with the DA-releasing psychostimulant drug, methylphenidate (MPH), involved three rat groups: prior i.v. administration of TP-22 (0.25 mg/kg), or vehicle (VEH), 90 min before MPH (5 mg/kg), was intended for modulation of conditioning to the white chamber (saline associated to the black chamber); control group (SAL) was conditioned with saline in both chambers. Prior TP-22 further increased the stimulant effect of MPH on locomotor activity. During the place-conditioning test, drug-free activity of TP-22+MPH subjects remained steadily elevated, while VEH+MPH subjects showed a decline. Finally, after a priming injection of TP-22 in MPH-free conditions, rats showed a high preference for the MPH-associated white chamber, which conversely had vanished in VEH-primed MPH-conditioned subjects. Overall, the interaction between MPH and pre-treatment with TP-22 seems to improve both locomotor stimulation and the conditioning of motivational drives to environmental cues. Together with recent studies, a main modulatory role of 5-HT7R for the processing of rewards can be suggested. In the present study, TP-22 proved to be a useful psychoactive tool to better elucidate the role of 5-HT7R and its interplay with DA in reward-related behavior.

## Introduction

The neurotransmitter serotonin (5-hydroxy-tryiptamine, 5-HT) is responsible for multiple physiological functions, including modulation of behavioral flexibility, cognition and memory processing, whereas its dysregulation has been often identified in many psychiatric disorders (Branchi, 2011; Sachs et al., 2015) as well as in addictive behavior (Müller and Homberg, 2014). Serotonergic drugs are widely used for therapy or abused as recreational drugs. Nevertheless, 5-HT multiple physiological roles are still under investigation and studies have often provided conflicting results, probably depending on different functioning of its many receptor subtypes. Among these subtypes, the serotonin 7 receptor (5-HT7R) was the last to be discovered, in 1993 (Bard et al., 1993). 5-HT7R is positively coupled to adenylate cyclase (AC) through activation of Gs, resulting in an intracellular increase of cAMP (Lovenberg et al., 1993; Ruat et al., 1993), and can also couple with G12, thus modulating neuronal morphology and increasing the neural network construction through activation of MMP-9 and Cdc42 (Bijata et al., 2017). 5-HT7R is broadly expressed in the central nervous system (CNS), with high concentrations in raphe, limbic areas, putamen and caudate nuclei, as well as in cortical regions (Leopoldo et al., 2011). 5-HT7R’s wide distribution in the CNS reflects its involvement in many functions (thermoregulation, circadian rhythm, sleep, learning, and memory). A dysregulation of 5-HT7Rs has been related to many neuropathological processes as well as to cognitive and mood dysfunctions, including anxiety, schizophrenia and depression (Kvachnina et al., 2005; Hedlund, 2009; Nikiforuk, 2015).

Assessing the exact implication of 5-HT7R in brain physiologic and pathologic mechanisms is complex because of the interaction between 5-HT7 and 5-HT1A receptors (Eriksson et al., 2012). These two receptor subtypes are localized in the same brain areas and exert opposite effects on the intracellular levels of cAMP. Unfortunately, most of the available ligands show a similar affinity for both receptors. Besides, 5-HT7 and 5-HT1A receptors can form both homo- and hetero-dimers (Renner et al., 2012). Functionally, the 5-HT7/5-HT1A hetero-dimerization decreases 5-HT1A receptor activity without affecting 5-HT7R-mediated signaling. In addition, hetero-dimerization is involved in the initiation of the serotonin-mediated 5-HT1A receptor internalization. A great advancement in this research area has been obtained with the identification of selective 5-HT7R antagonists and, more recently, of agonists.

Among these, the brain penetrant selective agonist LP-211 showed to be a suitable tool to elucidate the multiple functions of 5-HT7R *in vivo* (Romano et al., 2014). In previous studies, we investigated the modulatory effects of LP-211 on learning and memory processing, resulting in alterations of behavioral parameters (Beaudet et al., 2017; Carbone et al., 2018). In particular, activation of 5-HT7Rs, through LP-211 administration in rats, seems to favor exploration by enhancing visual consolidation and improving the ability to discriminate a familiar environment. Furthermore, LP-211 seems to strengthen the consolidation of emotional components of memory. These results suggest the potential use of LP-211 in the treatment of diseases that imply cognitive as well as emotional impairments, including depressive-like behavior (Zhang et al., 2015).

However, the activation of 5-HT7R alone gave contrasting results in several studies concerning the modulatory effect of this receptor on the depressive as well as the anxious-like behavior (Balcer et al., 2019; Maxwell et al., 2019). Indeed, while some research suggests that the blockade of the 5-HT7R is responsible for an antidepressant and anxiolytic effect (Lax et al., 2018), others prove that these same effects are unexpectedly also mediated by the activation of this receptor (Zhang et al., 2015). A possible explanation of this apparent inconsistency may rely on the synergistic role of both the serotonergic and the dopaminergic systems in modulating cognitive as well as emotional functions. Indeed, the exact direction of the serotonergic effects of drugs seems to depend on the simultaneous activation (or not) of the dopaminergic system. As just an example, the chronic administration of the selective serotonin reuptake inhibitor (SSRi) fluoxetine selectively upregulates dopamine (DA) D1-like receptors in the hippocampus of mice (Kobayashi et al., 2012); recent findings demonstrate that D1 receptors act as a pivotal mediator of the antidepressant action of this compound (Shuto et al., 2018). We underline therefore that, when studying the possible effects of a 5-HT7R-targeting drug, it is essential to also monitor for a modulatory dopaminergic intervention. Conversely, a modulatory serotonergic intervention can well be suggested, and addressed, for the well-known rewarding effects of psychostimulants.

In the present study, we tested the novel 5-HT7R agonist, TP-22, an arylpiperazine derivative structurally related to LP-211, the more thoroughly investigated agonist. TP-22 exhibited 5-HT7R agonist properties (Table 1) and improved *in vitro* metabolic stability as compared to LP-211 (half-life = 45 min and 15 min, respectively; Lacivita et al., 2016). TP-22 was able to stimulate neurite outgrowth in neuronal primary cultures in shorter time and at a lower concentration than LP-211, showing a comparable *in vivo* bio-distribution profile (brain C_max_ 515 ng/mL and 540 ng/mL, respectively; Lacivita et al., 2016; Modica et al., 2018). Starting from the evidence that the administration of methylphenidate (MPH) causes an upregulation of 5-HT7Rs (Adriani et al., 2006; Leo et al., 2009), and in light of the several findings that propose a physiological role for 5-HT in reward guided behavior (Broderick and Phelix, 1997; Luo et al., 2016; Fischer and Ullsperger, 2017), we formulated the hypothesis that a previous administration of a 5-HT7 agonist could have modulatory effects on the well-known rewarding and stimulant MPH effects.

**Table 1:** Binding affinity profiles (data taken from Lacivita et al., 2016).

	*K*_i_ (nM)				
Compound	5-HT7	5-HT1A	5-HT6	D2	Alfa-1
TP-22	25.5	771	614	522	6.6
LP-211	15	379	1,571	242	22.6

Therefore, we presently performed a conditioned place preference test (CPP), a common behavioral test for the associative rewarding effects of drugs in rodents. It is well known that psychostimulant vs. rewarding action can be dissected into D1-like vs. D2-like components (Stewart and Vezina, 1989). As such, we investigated here whether TP-22 also showed affinity towards either D1/D5 or D2/D3 dopaminergic receptors. Our goal was to further assess the role of the 5-HT7R in modulating the reinforcement process, presently triggered by MPH (Cummins et al., 2013). Ultimately, we sought to better understand the interplay between 5-HT and DA in reward-related behavioral adaptations.

## Materials and Methods

All experimental procedures have been approved by the ISS animal welfare survey board on behalf of the Italian Ministry of Health (formal license 937/2018-PR, to WA, veterinary surveillance by G. Panzini). Procedures were carried out in close agreement with the directive of the European Community Council (2010/63/EEC) and with the Italian Law guidelines. All efforts have made to minimize animal suffering and to reduce the number of animal used, according to the 3Rs principle.

### Experiment 1: The Multidose Pilot

#### Subjects, Rearing and Testing Conditions

Experimental subjects were 15 adult male (Wistar-Han) rats, born on April 2017 from the colony in our facility (>120 days old; average weight 420 g). Animals were placed at weaning in triplets within Plexiglas cages (33 × 13 × 14 cm), in an air-conditioned room (T 21° ± 1°C, relative humidity 60 ± 10%) with a 12 h dark-light cycle (lights turned on at 8.00 PM). Water and food (Altromin-R, A. Rieper S.p.A., Vandoies, Italy) were available *ad libitum*. The experiments were conducted inside the facility animal room to minimize the impact of transport to a novel testing room.

#### Locomotor Activity With TP-22

To assess the dose-related pharmacological effect of TP-22 on rats’ locomotor activity, we assigned animals to receive more than one injection following a Latin square design, to complete four dosage groups:

1. Control subjects, injected with vehicle (2% DMSO in saline solution, 200 μl/kg i.v.)
2. D25 subjects, injected with a dose of 0.25 mg/kg TP-22 i.v.
3. D12 subjects, injected with a dose of 0.12 mg/kg TP-22 i.v.
4. D06 subjects, injected with a dose of 0.06 mg/kg TP-22 i.v.

The home-cages were carefully placed on a cart, the three homemate animals were weighed, injected and gently placed individually in new home-cage-like Plexiglas cages with clean sawdust, which were immediately positioned in a recording rack. The experiment was designed so that each homemate out of a triplet was randomly assigned to receive more than one of the four planned doses (vehicle, 0.25 mg/kg, 0.12 mg/kg, 0.06 mg/kg) on separate days in a counterbalanced order. Intravenous (i.v.) injection was selected as a route of administration of TP-22 since it causes a rapid onset of action, bypassing the first-pass gastro-intestinal and hepatic metabolism. Furthermore, through i.v. administration, the possible visceral side effects (due to the presence of 5-HT receptors in the gastro-intestinal trait) have been avoided. After injection, the rats were monitored for a total of 24 h, of which only the first 4 h after injection were analyzed.

#### Experimental Apparatus

A recording rack was used to continuously monitor for locomotion. The ActiviScope system^®^ is an automatic device, with small passive infrared sensors placed over the top of each home-cage (ActiviScope; TechnoSmart, Rome, Italy)^1^. Locomotor activity cycle was measured as number of infrared interruptions caused by the movement of the rat (i.e., the infrared source) under the sensor (counts taken at 20 Hz, i.e., up to 20 counts per second). Data were recorded by a computer with dedicated software. Scores were automatically divided into 10-min intervals and then further grouped three by three to obtain 30-min bins. The access of authorized personnel to the animal room was not restricted and followed the routine schedule.

### Experiment 2: Conditioned Place Preference With MPH and TP-22

#### Subjects, Rearing and Testing Conditions

Experimental subjects were 18 adult male (Wistar-Han) rats born on April 2017 from the colony in our facility (>240 days old; average weight 560 g). Animals were placed at weaning in triplets within Plexiglas cages (33 × 13 × 14 cm), in an air-conditioned room (T 21° ± 1°C, relative humidity 60 ± 10%) with a 12 h dark-light cycle (lights turned on at 8.00 PM). Water and food (Altromin-R, A. Rieper S.p.A., Vandoies, Italy) were available *ad libitum*. The experiments were conducted inside the facility animal room to minimize the impact of transport to a novel testing room.

#### Experimental Apparatus

The experimental apparatus used for the CPP test is a Black/White Box (BWB; Adriani et al., 2012), i.e., a Plexiglas box with smooth walls and floor (70 × 30 × 35 cm) composed of two environments separated by a central gray wall placed at a distance of about 35 cm from end walls. The walls on the longer sides are gray whereas those on the short margins can be distinguished by black or white color. To make the two environments more recognizable, we added additional visual cues: three horizontal white stripes to the black wall and three vertical black stripes to the white wall. On the central gray wall there is a door with an easily removable panel (partition), allowing the experimental subjects to pass (or not) from one compartment chamber to the other, when required.

On both longer sides of the box, there are two aluminum bars equipped with eight photocells connected by cables to a computer. The software in use is *Cage controller 1.27 for Dark Light for Rat and Mouse*^®^ (PRS, Rome, Italy)^2^, that allows to score for each subject:

1. Motor activity (beam interruptions per second) in either chamber
2. Time spent in each chamber (both forepaws and hindpaws in a same chamber)
3. Transitions (number of times a subject crosses the door between the two chambers)

Data were divided into 300 s intervals (bins).

#### Experimental protocol

This test was carried out under dim light and required nine not consecutive days divided into four steps (Figures 2–4):

- *Day 1, initial preference test*. The spontaneous place preference of experimental subjects was tested in drug-free conditions. They were initially placed in the black chamber (chosen as starting room) and central door was open during the whole test (15 min). The triplet of rats residing in each home cage was tested at the same time.
- *Days 2–7, drug conditioning*. On odd days all 18 subjects were injected with saline solution only and, after injection, immediately placed in the black chamber. The central door was closed and animals were forced to remain in the black chamber for the duration of the session (25 min) in order to associate the lack of any pharmacological effect with this environment. On even days subjects were injected with only saline, 2% DMSO+MPH or TP-22+MPH according to groups described below and, after the last injection, immediately placed in the white chamber. The central door was closed so that animals were forced to stay in the white side during all the 25-min session, in order to associate the pharmacological effects with this environment. This procedure was repeated three times, alternating saline day and drugs days.
- *Day 8, post-conditioning preference test*. The post-conditioning place preference of experimental subjects was tested in drug-free conditions. This session was conducted in exactly the same way as the initial preference test (see above). Rats were placed in the black chamber as starting room and allowed to freely access and explore both environments.
- *Day 9, post-conditioning preference test with priming*. To test the acute effect of a TP-22 pre-treatment on the place preference, after a week only the MPH conditioned subjects were tested again. The rats were injected respectively with either DMSO 2% or TP-22 and, 1 h and a half after, they were placed in the black start room for the 15-min free-choice task, in MPH-free conditions.

**Figure 1:**
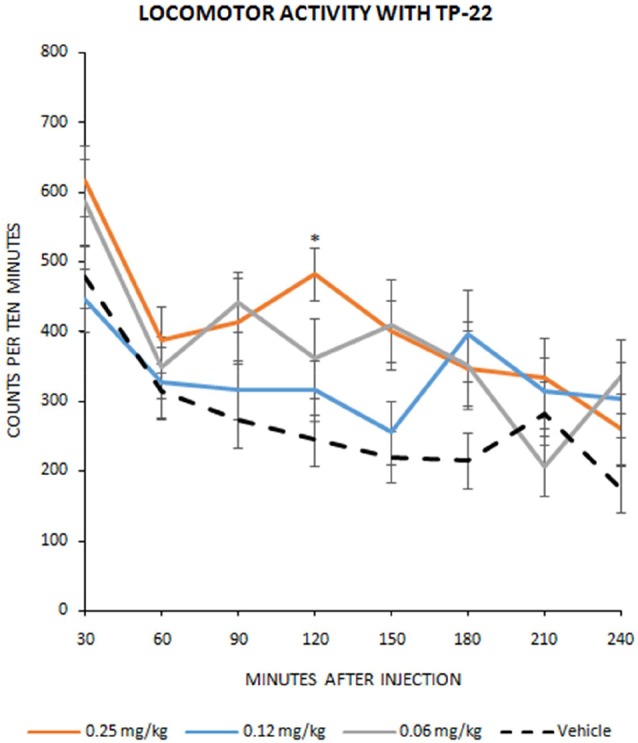
Locomotor activity with TP-22. Number of infrared interruptions (20 Hz sensors) in the home-cage, during eight 30-min bins following injection (mean ± SEM). Dose-related pharmacological effect on home-cage locomotor activity were assessed on 15 Wistar rats in a Latin square design, to complete four dosage groups: vehicle group, 0.06 mg/kg TP-22 i.v., 0.12 mg/kg TP-22 i.v., 0.25 mg/kg TP-22 i.v. (counterbalanced across days). Significant difference in response emerged between 0.25 mg/kg dose and vehicle, especially for the time-point of 90–120 min after the injection. *P-value < 0.05.

**Figure 2:**
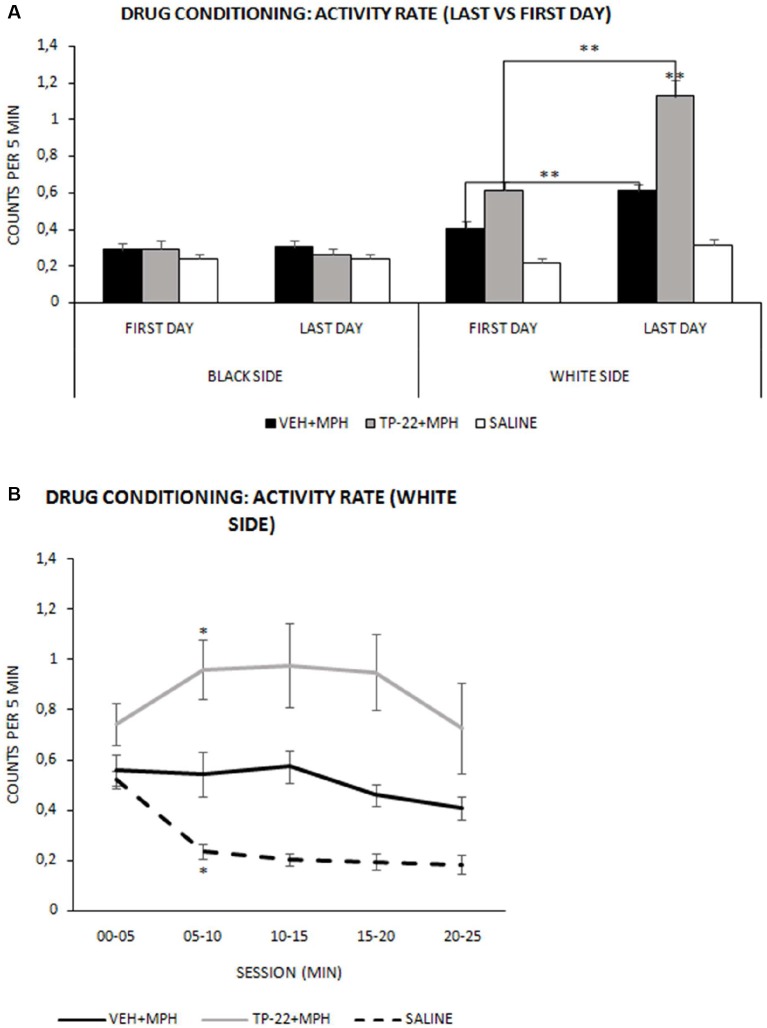
Drug conditioning phase: activity rate. Mean (± SEM) activity rate measured as number of beam crossing per second during the 25-min session (VEH+MPH *n* = 6; TP-22+MPH *n* = 6; SAL *n* = 6). Activity rates of subjects were recorded during the first and last drug conditioning sessions for both black (saline-associated) and white (drug-associated) chambers. **(A)** TP-22+MPH group displayed a significantly enhanced locomotor activity during the last conditioning session compared to VEH+MPH subjects as well as to the first session (*P* < 0.01) only in the white side (associated with drug administration). **P-value < 0.01. **(B)** Last session’s activity rate (in the white side) divided into five 300-s intervals. TP-22+MPH subjects showed a significantly increasing locomotor activity, specifically between 05 and 10 min; VEH+MPH subjects displayed a constant activity for the entire session and control group showed net decrease in locomotor activity during the last conditioning session. *P-value < 0.05.

**Figure 3:**
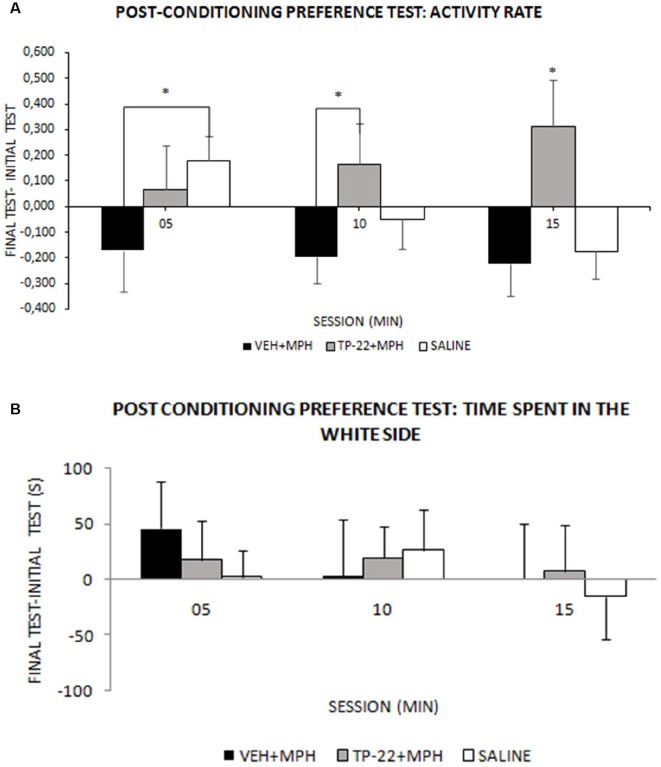
Post-conditioning preference test. Wistar rats were subjected to the conditioning (as illustrated in Figure 2), then to a post-conditioning preference test in drug-free conditions and results were compared by subtraction with the initial preference test. **(A)** Activity in either chamber: mean activity rate (± SEM) measured as number of beam crossing per second (VEH+MPH *n* = 6; TP-22+MPH *n* = 6; SAL *n* = 6). Activity rate of TP-22+MPH subjects was significantly higher, compared to both VEH+MPH (05–10 and 10–15 min) and to saline injected rats (10–15 min), and also continued to increase over time. VEH+MPH locomotor activity (post-conditioning preference test minus the initial preference test) was stably negative during the entire session. *P-value < 0.05. **(B)** Time (s) spent in the white chamber: preference for the white chamber during the 15 min test session, measured into three 300-s intervals (VEH+MPH *n* = 6; TP-22+MPH *n* = 6; SAL *n* = 6).

**Figure 4:**
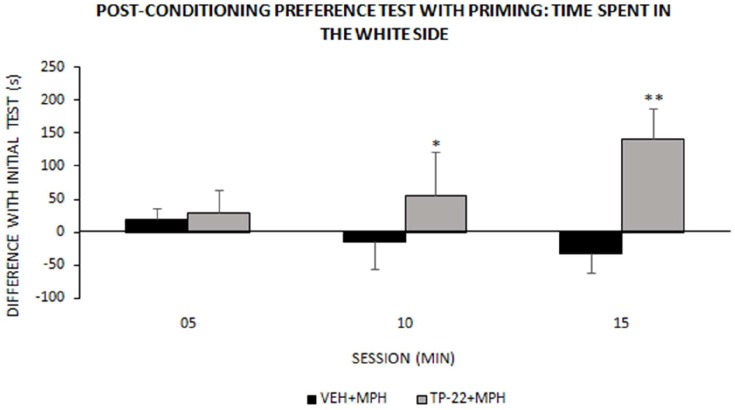
Post-conditioning preference test with priming: time spent in the white side. This test occurred around one week after place-conditioning test (illustrated in Figure 3). Time (s) spent in the white chamber (mean ± SEM) during the 15 min test session, measured into three 300-s intervals (VEH+MPH *n* = 6; TP-22+MPH *n* = 6). Subjects were subjected to a preference test performed 1 h and a half after a “priming” injection: TP-22+MPH rats received TP-22 (0.25 mg/kg i.v.) whereas VEH+MPH subjects were injected with DMSO 2% in saline; animals received no further injection before being placed in the apparatus, for a MPH-free choice. Time spent in the white side by TP-22+MPH rats was gradually increasing over time, denoting unexpected attraction; on the contrary, time spent in the white side by VEH+MPH subjects gradually decreased during the session. *P-value < 0.05, **P-value < 0.01.

#### Conditioned CPP Drug Conditioning, Place Preference With MPH and TP-22 Modulation: Drug Conditioning

Home-cages were carefully placed one by one on a cart adjacent to the three experimental apparatuses. Rats residing in triplets within each home-cage were treated and tested at the same time. Animals were individually injected with intravenous infusions (200 μl/kg): TP-22 at a dose of 0.25 mg/kg or vehicle, MPH (5 mg/kg), or saline solution 0.9%. Tests were conducted during the dark cycle between 9:30 AM and 14:30 PM. In this experiment, a pre-treatment with TP-22 was administered for assessing its modulatory effects on the place conditioning with MPH and resulting preference. To study modulation by TP-22, subjects received one administration of TP-22 (or vehicle) 1 h and a half before MPH and the start of the white-chamber session. Three experimental groups were therefore formed (see Table 2):

1. Control subjects (*N* = 6), injected with saline solution 0.9% immediately before the start of the session in white chamber (SAL).
2. Subjects pre-injected with vehicle (DMSO at 2% in saline, *N* = 6) 1 h and a half before, then with MPH dissolved in saline solution immediately before the start of the session in white chamber (VEH+MPH).
3. Subjects pre-injected with TP-22 dissolved in vehicle (DMSO at 2% in saline, *N* = 6) 1 h and a half before, then with MPH dissolved in saline solution immediately before the start of the session in white chamber (TP-22+MPH).

**Table 2:** Experimental groups.

SALINE GROUP	VEH+MPH GROUP	TP-22+MPH GROUP
Initial place preference test in drug free conditions.	Initial place preference test in drug free conditions.	Initial place preference test in drug free conditions.
Drug conditioning: saline in both black (3x) and white (3x) chambers.	Drug conditioning: saline in black chamber (3x), previous VEH then MPH in white chamber (3x).	Drug conditioning: saline in black chamber (3x), previous TP-22 then MPH in white chamber (3x).
Post conditioning preference test in drug free conditions.	Post conditioning preference test in drug free conditions.	Post conditioning preference test in drug free conditions.
	1 Week after	
	Post conditioning preference test in MPH free conditions with priming (VEH).	Post conditioning preference test in MPH free conditions with priming (TP-22).

Timing and dose of TP-22 were selected based on the previous pilot experiment (Experiment 1), in which TP-22 showed its maximum effect on locomotor activity after 1 h 30 min at the dose of 0.25 mg/kg.

After exposure to the session in the white chamber, each subject was gently replaced in his own home-cage. On alternate days, the same subjects were exposed to the black chamber following saline injection (“biased” CPP).

### Radioligand Displacement Assays at the Human Dopamine D2 and D3 Receptors

Cell culture and membrane preparations were performed with slight modifications to Sokoloff et al. (1992). Briefly, CHO cells containing the D2-short receptor were cultured in DMEM F12 (supplemented with 10% FBS, and 1% penicillin/streptomycin). CHO cells transfected with D3 receptor were cultured in DMEM containing 1% glutamine and 10% FBS. After reaching confluence, the cells were collected in PBS buffer and centrifuged (3,000× *g*, 10 min, 4°C). The pellet was resuspended in binding buffer (1 mM MgCl_2_, 1 mM CaCl_2_, 5 mM KCl, 120 mM NaCl and 50 mM Tris, pH 7.4), disrupted and centrifuged at 23,000× *g* for 30 min (4°C). The resulting pellet was stored in binding buffer at −80°C for further use.

Radioligand displacement assays were performed as reported previously (Frank et al., 2018). Briefly, membrane preparations described above were co-incubated with [^3^H]spiperone (0.2 nM) and the test ligand. Non-specific binding was measured with haloperidol (10 μM). Concentrations required to inhibit 50% of radioligand specific radioligand binding (IC_50_) were determined by using six to nine different concentrations (0.01 nM–10 μM) of the drug studied in two or three experiments with samples in duplicate/triplicates. Apparent inhibition constant (*K*_i_) values were determined by non-linear least-squares fitting and equation “one site competition” using Prism 7^®^ (GraphPad Software Inc., San Diego, CA, USA). All statistical operations were performed on the p*K*_i_ values and converted afterward to mean *K*_i_ values and the 95% confidence interval.

### Radioligand Displacement Assays at the Human Dopamine D1 and D5 Receptors

CHO cells stably expressing the human DA D1 and or D5 receptor were washed and collected with PBS buffer. Membrane preparations were obtained as described previously (Bautista-Aguilera et al., 2017). Membrane preparations (20 and 10 μg/well in a final volume of 0.2 ml binding buffer for D1R and D5R, respectively) were incubated for 120 min with [^3^H]-SCH23390 (0.3 nM) and the test ligand. Nonspecific binding was measured with fluphenazin (100 μM). Concentrations required to inhibit 50% of radioligand specific binding (IC_50_) were determined by using 6–9 appropriate concentrations of the drug studied in two or three experiments with samples in duplicate/triplicates. Apparent inhibition constant (*K*_i_) values were determined by non-linear least-squares fitting and equation “one site competition” using Prism 7^®^ (GraphPad Software Inc., San Diego, CA, USA). All statistical operations were performed on the p*K*_i_ values and converted afterward to mean *K*_i_ values and the 95% confidence interval.

### Dopamine D1 Receptor Gs-Mediated cAMP Accumulation Assay

The agonist and antagonist properties of TP-22 against the human D1 receptor were evaluated in functional assays performed at Eurofins^3^ using CHO cells expressing D1 and according to previously reported protocols (Zhou et al., 1990). To assess the agonist properties, TP-22 was tested using eight different concentrations in two experiments with samples in duplicate. Cellular agonist effect was calculated as % of control response to the reference agonist DA (EC_50_ = 24 nM). To assess the antagonist properties, cells were stimulated with DA (125 nM) and the effect of TP-22 on cAMP production was assessed using eight different concentrations in two experiments with samples in duplicate. The antagonist effect was calculated as % inhibition of reference agonist response. The standard D1R antagonist SCH 23390 was tested as reference compound (IC_50_ = 2.9 nM, K_b_ = 0.45 nM).

### Statistics

#### Behavioral Data

##### Experiment 1

Data were analyzed using StatView II^®^ (Abacus Concepts, CA, USA) and were processed by analysis of variance (ANOVA). Analysis was carried out by a split-plot 4 × 8 model (average *n* = 10/11 per group) with two independent variables: treatment (four levels: VEH, D25, D12, D06), and time (eight levels: 30-min bins from 4 h of registration).

##### Experiment 2

Data (displayed as mean ± SEM) were analyzed using StatView II^®^ (Abacus Concepts, CA, USA) and were processed by ANOVA. For test and priming (days 8 and 9), analysis was carried out by a split-plot 3 × 3 × 2 model with three independent variables: treatment (three levels: SALINE, VEH+MPH and TP-22+MPH), time (3 levels: 05, 10, 15 min bins), and side (two levels: black side and white side). The dependent variable was calculated as final preference day minus initial preference day. Drug conditioning design implied the same independent variables with addition of a two-level day (first vs. last) factor.

Level of significance was set at *P* < 0.05; significant trends at 0.10 < *P* < 0.05 were also considered whenever effects were then confirmed by *post hoc* analysis. Multiple *post hoc* comparisons were run by Tukey HSD test, which is protected against the false positives and may be used even on non-significant ANOVA effects. Sample size was calculated before starting the experiment: from values of *P* = 0.05 and power of 0.80 with expected increases of 30% in all variables, the appropriate groups should have *N* = 6 each. Two experimental subjects showed an overtly abnormal behavior including lack of interest in environmental exploration and extreme inactivity during the entire duration of the experiment. After performing an interquartile range test, they were identified as outliers and excluded from the analysis.

#### Dopamine D1 Receptor Gs-Mediated cAMP Accumulation Assay

The effect on cAMP accumulation of TP-22 was determined as a percent of control agonist response or inverse agonist response and as a percent inhibition of control agonist response. The EC_50_ values (concentration producing a half-maximal response) and IC_50_ values (concentration causing a half-maximal inhibition of the control agonist response) were determined by non-linear regression analysis of the concentration-response curves generated with mean replicate values using Hill equation curve fitting. Analysis was performed using custom software developed at Cerep (Hill software) and validated by comparison with data generated by the commercial software SigmaPlot^®^ (SPSS Inc., USA). For the antagonists, the apparent dissociation constants (Kb) were calculated using the modified Cheng–Prusoff equation.

## Results

### Experiment 1: The Multidose Pilot

#### Locomotor Activity

Dose-related pharmacological effect on locomotor activity, after treatment with TP-22, showed a significant trend (*F*_(3,38)_ = 2.612, *P* = 0.0653), confirmed by *post hocs* (see below), whereas interaction between time and treatment did not show significance (*F*_(21,266)_ = 0.769, *P* = 0.7570). *Post hoc* analysis, performed with the Tukey HSD test, displayed a significant difference in dose response between D25 (0.25 mg/kg) and vehicle (*P* < 0.05) especially for the time-point of 120 min after the injection. D12 (0.12 mg/kg) and D06 (0.06 mg/kg) did not seem to differ from the vehicle control (Tukey threshold = 128.5; *df* = 38; *k* = 3; Figure 1).

### Experiment 2: Conditioned Place Preference With MPH and TP-22

#### Drug Conditioning Days

We recorded and analyzed activity rates for all three groups during the first and last drug conditioning sessions. Regarding the treatment, overall analysis displayed a significantly increased locomotor activity (*p* < 0.05) in both TP-22+MPH and VEH+MPH subjects compared to control subjects (saline solution; Treatment: *F*_(2,13)_ = 16.076, *P* = 0.0003). There was also a significant locomotor activity increase in rats who received TP-22 compared to rats receiving vehicle 1 h and a half before the MPH injection (VEH+MPH subjects; data not shown).

It should be noted that locomotor activity was increased only in the white side (drug associated), as expected. Indeed, both TP-22+MPH and VEH+MPH subjects showed in this chamber an enhanced activity (*P* < 0.01) compared to the black (and saline-associated) one (Side*Treatment: *F*_(2,13)_ = 31.439, *P* < 0.0001). Hence, for both these groups locomotor activity appeared higher on the days of drug administration (white chamber) compared to those of saline administration (black chamber), whereas control subjects did not show any difference. Moreover, on days with drug administration, locomotor activity of TP-22+MPH subjects was significantly increased (*P* < 0.01) compared both to VEH+MPH subjects and control group. Activity of VEH+MPH group was also significantly enhanced compared to control group (*P* < 0.01). On the contrary, on days with black-side saline administration, locomotor activities of all the three groups appeared superimposable (data not shown).

For both TP-22+MPH and VEH+MPH groups analysis displayed a significantly enhanced locomotor activity during the last conditioning session compared to the first one (*P* < 0.01) only in the white side (associated with drug administration), denoting a presumable sensitization to the drug effect: of course, there were no such differences for control group in both sides of the apparatus (Side*Day*Treatment: *F*_(2,13)_ = 11.367, *P* = 0.0014). Notably, TP-22+MPH subjects showed a significantly higher locomotor activity than VEH+MPH ones, in particular during the last conditioning session (*P* < 0.01; Figure 2A). After last drug administration (white side) TP-22+MPH subjects showed a significantly increased locomotor activity specifically between minutes 05 and 10 (*P* < 0.05), whereas MPH subjects displayed a constant activity for the entire duration of the conditioning session (white side: Time*Treatment *F*_(8,52)_ = 5.687, *P* < 0.0001). On the contrary, control group showed a clear and significant decrease in locomotor activity between minutes 05 and 10, as expected (*P* < 0.05; Figure 2B).

#### Post-conditioning Preference Test

We recorded and analyzed time spent in each chamber, activity rate and transitions for all three groups during the post-conditioning preference test, in drug-free conditions. Time spent in the white side after drug conditioning did not seem significantly different from the initial preference test and analysis did not show any significant difference between the three experimental groups except for a slight preference for the white side displayed by the VEH+MPH in the first 05 min (Time*Treatment: *F*_(4,26)_ = 0.356, *P* = 0.8374; Figure 3B). Nevertheless, activity rate and transitions showed an interesting profile. The difference in VEH+MPH locomotor activity between the post-conditioning preference test and the initial preference test was negative during the entire session, whereas activity rate of TP-22+MPH subjects was positive and significantly higher compared to both VEH+MPH (05–10 and 10–15 min, *P* < 0.05) and to saline-injected rats (10–15 min, *P* < 0.05); locomotor activity of TP-22+MPH rats also continued to increase over time (Time*Treatment: *F*_(4,26)_ = 2.663, *P* = 0.0550), an effect particularly evident in the white side (Side*Time*Treatment: *F*_(4,26)_ = 3.190, *P* = 0.0294; Figure 3A). Locomotor activity of saline-injected subjects gradually diminished over time, as expected.

Even transitions between the two environments were gradually decreasing for saline-injected subjects as well as for VEH+MPH rats, whereas TP-22+MPH subjects crossed the door dividing the two environments with increasing frequency over time (Time*Treatment: *F*_(4,26)_ = 2.841, *P* = 0.0445). *Post hoc* analysis displayed a significant difference between TP-22+MPH and VEH+MPH group in the time interval between 05 and 10 min, whereas there were no differences between both these groups and saline controls (*P* < 0.05; data not shown).

#### Post-conditioning Preference Test With Priming

We recorded and analyzed time spent in each chamber, activity rate and transitions for TP-22+MPH and VEH+MPH group in a preference test performed 1 h and a half after a “priming” injection: the latter was TP-22 for TP-22+MPH subjects and DMSO 2% for VEH+MPH group. The difference in time spent in the white side between the “primed” preference test and the initial preference test was the dependent variable: it was positive for TP-22+MPH subjects whereas VEH+MPH ones spent less time in the white side compared to the initial preference test (Treatment: *F*_(1,8)_ = 4.883, *P* = 0.0581).

Furthermore, time spent in the white side by TP-22+MPH rats was gradually increasing over time whereas VEH+MPH subjects gradually decreased the time spent in the white side during the session. There was indeed a significant trend for Time*Treatment (*P* = 0.0963) and *post hoc* analysis showed that time spent in the white side by TP-22+MPH subjects during the time interval between 05 and 15 min was significantly higher compared to VEH+MPH group (*P* < 0.05), in particular during the last 5-min (*P* < 0.01; Figure 4). Regarding activity rate, there were no significant differences between the two groups. There were no significant differences in transitions too.

### Affinity at Dopamine D1, D2, D3 and D5 Receptors

The radioligand displacement assays indicated that TP-22 interacted differently with DA receptor subtypes. In fact, the compound displayed considerable affinities at human DA D1 (*K*_i_ = 3.93 nM) and D5 (*K*_i_ = 16.9 nM) receptors, whereas it showed much lower affinities at human DA D2 (*K*_i_ = 1127 nM) and D3 (*K*_i_ = 1512 nM) receptors (Table 3).

**Table 3:** Dopamine receptor subtypes affinities as measured by radioligand binding experiments.

*K*_i_ (nM) [95% CI] (nM) ± SEM			
D1 receptor	D2 receptor	D3 receptor	D5 receptor
3.93	1,127	1,512	16.9
[1.11; 14.0]	[605; 2,098]	[1,092; 2,093]	[10.8; 26.7]
3.93 ± 0.7	1,127 ± 130	1,512 ± 92	16.9 ± 2.1

*Data is expressed as means with the corresponding 95% confidence interval*.

### Functional Activity at Dopamine D1 Receptor

The evaluation of the DA D1 receptor Gs-mediated cAMP accumulation indicated that TP-22 behaved as an antagonist. In fact, TP-22 alone was not able to induce cAMP accumulation showing less than 25% effect at the highest validated testing concentration. In the same assay, the standard agonist DA showed EC_50_ value of 24 nM. Instead, TP-22 behaved as a competitive antagonist at D1 receptor being able to dose-dependently antagonize the agonist response with low and sub-micromolar potency (IC_50_ = 0.97 nM; *K*_b_ = 0.16 nM).

## Discussion

MPH, commonly prescribed for the treatment of Attention-Deficit/Hyperactivity Disorder (ADHD), is a psychostimulant drug whose mechanism of action is indirect DA agonism: MPH inhibits the DA transporter protein, increasing the DA concentration in the synaptic cleft (Volkow et al., 2002). Its psychostimulant vs. rewarding action can be dissected into D1-like vs. D2-like components (Stewart and Vezina, 1989). In the present study, we found that prior administration of the selective 5-HT7R agonist/D1-like receptor antagonist TP-22, at a dose of 0.25 mg/kg i.v., increased the stimulant effect of MPH on locomotor activity during the subsequent acute and subchronic administration. Furthermore, after the conditioning phase, 3 days after the last drug administration, the activity of TP-22+MPH injected subjects remained steadily elevated, whereas the MPH-only injected subjects showed a sharp decline in activity compared to the saline vehicle controls. Finally, after a priming injection of TP-22 in MPH-free conditions, rats showed a higher preference for the MPH-associated white chamber than the vehicle-primed subjects. Overall, compared to the administration of MPH alone, the interaction between MPH and prior TP-22 seems to improve the conditioning of motivational drives to environmental cues, suggesting a main modulatory role of 5-HT7R in processing of rewarding power of psychostimulants.

In the first experiment, we characterized a range of TP-22 dosages by means of a recording rack, and found that the 0.25 mg/kg dose was the most effective in altering locomotor activity: the observed increase, although marginally significant in the ANOVA, was however fully significant by Tukey *post hoc* analysis. The maximum effect was reached an hour and a half after acute drug administration.

Subsequently, we performed a CPP test with MPH and its modulation by TP-22 at the selected dose. An initial preference test was run in order to assess the spontaneous locomotion and place preference in drug-free conditions. During the drug conditioning phase, we found that the activity rate of TP-22 pre-injected rats resulted significantly higher than both other groups, suggesting a potentiation when compared to the MPH-only injected subjects.

A possible explanation of this excitatory effect may be an enhanced drug-induced stimulation. In other words, we may propose enhanced post-synaptic effects, due to prior activation of 5-HT7R, related to (even unchanged) DA release by MPH. As a matter of fact, different studies have demonstrated that 5-HT-releasing drugs, such as 3,4-methylenedioxy-methamphetamine (MDMA), are experienced as inducing a more positive mood by humans, even compared to high doses of amphetamine (Camí et al., 2000; Tancer and Johanson, 2003; Carhart-Harris et al., 2015). On the other hand, selective 5-HT releasers that spare DA are not experienced as pleasurable by humans (Tancer and Johanson, 2003). Furthermore, the 5-HT7R selective antagonist, SB-269970, significantly attenuated amphetamine-induced hyperactivity in mice and rats (Galici et al., 2008; Waters et al., 2012).

Another relevant feature of TP-22 is that the affinity for D2 and D3 DA receptors (Tables s 1, 3) is far lower than that for 5-HT7R and D1-like receptors. A few considerations shall be put forward. First, we presently found that TP-22 has great affinity to D1 receptors at which it acts as an antagonist. According to previous literature, D1 antagonists usually inhibit the MPH effect on locomotor activity (Claussen et al., 2015). Therefore, hyperlocomotion induced by TP-22 cannot rely on its D1 antagonism and is likely due to its action onto 5-HT7R. However, the dosage of TP-22 producing a peak of locomotion in the multidose pilot (Experiment 1) was quite low, so that the engagement of DA system through direct interaction of TP-22 with such D1 DA receptors might be questioned. In any case, potentiation of MPH stimulation was not caused by an additive and direct D1 receptor activation after TP-22 acute administration. Such notions may confirm a modulatory role of 5-HT7R, recruited by TP-22, on the stimulant effect elicited by DA-releasing drugs such as MPH.

In the third step, we examined place preference in drug-free conditions again. MPH-subjects spent slightly more time in the white (drug-associated) chamber during the first 5-min. However, interesting results emerged concerning locomotor activity. Regardless of conditioning effect, MPH-subjects had a clear-cut decline in activity when exposed in drug-free to the environment. This was, probably, due to conditioned inhibitory effects on DA neuronal activity: this resembles the well-known “down” elicited by psychostimulants like MPH (Shi et al., 2000; Dela Peña et al., 2018). Indeed, MPH, as well as other amphetamine-like psychostimulants, may inhibit DA neuron firing by increasing extracellular DA and by activating DA D2 autoreceptors and long-loop feedback pathways. On the contrary, activity and transitions of TP-22+MPH injected subjects remained steadily elevated and continued to increase over time during the post-conditioning test, particularly in the white side. These results may suggest a long-lasting enhancement of brain reward activity, due to 5-HT7R action of TP-22. As an additional possibility, the likely occupancy and incomplete blockade of D1 receptors, by TP-22 subchronic exposure, may well prevent the aforementioned DA depletion. Contrarily than VEH+MPH rats, and similarly to a “rebound” phenomenon, a receptor upregulation may have occurred in TP-22+MPH rats, leading to a further increase in locomotor activity during the post-conditioning test session. On the same reasoning, D1 antagonism possibly showed by TP-22 may be partly responsible for the enhanced sensitization to the MPH stimulant effects, displayed by the TP-22 pretreated subjects during the conditioning days (subchronic administration).

The fourth step was a place preference test with priming: this was to assess the eliciting effect of a TP-22 pre-injection. Notably, the TP-22 pre-injected subjects displayed a clear and increasing preference toward the white environment, compared to the vehicle-primed subjects. The latter, conversely, showed a slight preference for the black chamber, denoting that their slight preference for MPH-associated chamber had already vanished. A higher expectation for reward, elicited by just approaching the white chamber, seems to be the behavioral driving force of TP-22-primed subjects.

LP-211, as well as other 5-HT7R agonists, showed in several studies to modulate the construction of neural networks, hence improving the long-term memory (Meneses et al., 2015; Shahidi et al., 2018). TP-22 is able to stimulate neurite outgrowth in neuronal primary cultures in a shorter time and at a lower concentration than LP-211 (Lacivita et al., 2016). Overall, as already highlighted for LP-211, our results suggest that TP-22 may enhance the consolidation of emotional components of memory, causing a stronger association to develop between the environment and the MPH-driven hedonic experience. Alternatively, or in parallel, TP-22 may potentiate the consolidation of visual components (Carbone et al., 2018): hence, the MPH-associated white chamber could be better recalled. The encoding of novel visuo-spatial information also involves activation of DA D1/D5 receptors, as demonstrated in several studies performed on rats (see Lemon and Manahan-Vaughan, 2006, 2012; Hagena and Manahan-Vaughan, 2016).

Nevertheless, the D1-like receptor antagonism, possibly shown by TP-22, seems to exclude a direct involvement of dopaminergic pathways. According to previous literature, D1/D5 antagonists usually inhibit the acquisition of drug-related incentive memories, in particular for cocaine CPP memories (Kramar et al., 2014); on the other hand, D1 receptor antagonism reduces compulsive-like reward seeking and restores behavioral flexibility (Barker et al., 2013): as such, TP-22 priming injection is unlikely to trigger an overt seeking for MPH effects. In this line, activation of 5-HT neurons promotes patience to wait for future reward (Miyazaki et al., 2012, 2014; Liu et al., 2014). The theory assumes that activation of 5-HT neurons increases the subjective confidence of reward delivery (Li et al., 2016; Miyazaki et al., 2018). Therefore, after repeated TP-22/MPH pairings established a CPP, an acute 5-HT7R activation alone (as witnessed by our “priming” test) seems to trigger a hedonic attraction for the white MPH-conditioned chamber, in that it potentiates the expectation of experiencing again a reward therein. Since TP-22 priming injection *per se* did not massively affect locomotor activity nor transitions, it is confirmed that TP-22 alone is only a weak stimulant; conversely, in the TP-22+MPH group during the pairing phase, the stimulation was nearly double compared to MPH alone: effects on these parameters become likely additive.

### Implications for Serotonergic Modulation of Reward

Several theories have been proposed to explain the implication of 5-HT neurons in behavioral modulation, i.e., the “punishment” theory (Soubrié, 1986; Dayan and Huys, 2009); the “behavioral inhibition theory” (Miyazaki et al., 2011, 2012); the “mood” theory (Daw et al., 2002; Savitz et al., 2009). Many authors have reported modulatory effects on reward processing by manipulating central 5-HT levels (Roiser et al., 2006; Tanaka et al., 2007; Seymour et al., 2012) while others have proved that the serotonergic system is implied both in punishment and reward processing (Palminteri et al., 2012; Worbe et al., 2016; Scholl et al., 2017). Recent physiological, neuropathological, and optogenetic studies suggest an interpretation that seems to reconcile the different and controversial results so far achieved (Fischer and Ullsperger, 2017). The origin of most of the forebrain serotonergic innervation is the dorsal raphe nucleus (DRN), which is functionally interconnected with the ventral tegmental area (VTA). Recently, novel cell-type specific tracing techniques allowed to describe more precisely the structural connectivity between DRN and VTA. It was found that DRN projects to VTA mainly *via* glutamatergic, but additionally via 5-HT co-releasing neurons (McDevitt et al., 2014; Qi et al., 2014). Tonically stimulating 5-HT neurons in the DRN produces a weak reinforcing effect (Liu et al., 2014; Fonseca et al., 2015). Therefore, while DA neurons are critical for driving motivation (i.e., wanting), 5-HT neurons of DRN may play a major role in other aspects of rewards, such as consolidation of positive emotion and predictions *via* context evaluation. Hence, DA and 5-HT could provide a combined reward signal, whereas its dissociation (i.e., 5-HT in absence or reduction of DA) may encode punishment (see Fischer and Ullsperger, 2017).

Some recent studies can shed light on the persistent state of excitement showed by TP-22+MPH animals. Several animal and human studies demonstrate that 5-HT not only transfers information on short timescales but also acts over protracted timescales of days and weeks (Cohen et al., 2015; Luo et al., 2016; Scholl et al., 2017) leading to changes in plasticity, as also proven through 5-HT7R activation (Jitsuki et al., 2011; Bijata et al., 2017), and in particular by administration of TP-22 (Lacivita et al., 2016). Hence, the previous activation of 5-HT7R combined with the subsequent administration of MPH may have changed the perception of the white environment. TP-22 was enhancing the possible consolidation of information about the net benefit of the current context (white chamber; Luo et al., 2016).

We have been studying such interaction during a decade. Evidences, emerged from our previous studies, suggested an interplay between DA neurotransmission and 5-HT7R (Adriani et al., 2006; Leo et al., 2009). Indeed, MPH administration to adolescent rats, besides a reduction of basal behavioral impulsivity, produced a marked and persistent increment of 5-HT7R expression, denoting that MPH-induced effects could have been mediated, at least in part, by 5-HT7R modulation. Our present findings suggest that MPH, combined with activation of 5-HT7R and possibly with D1/D5 antagonism (besides additive modulatory effect on DA-induced arousal), could be strongly involved in drug conditioning mechanisms, switching the MPH-free preference by rats to the previously reward-associated environment.

## Conclusion

Further investigation is needed to thoroughly assess the role of 5-HT7Rs in punishment/reward-guided learning, nevertheless, the present outcomes suggest a direct involvement of 5-HT7R in modulating reward features, such as conditioned locomotion and priming of drug seeking. Beyond present results, activation of 5-HT7R combined with the D1/D5 modulation may lead to an improvement in behavioral flexibility, therefore to a better adaptation to adverse situations (Ruocco et al., 2014).

The great potential of targeting together the 5-HT7R and D1R is noticeable: a putative application may be to trigger the confidence for rewarding experiences in the presence of salient environmental cues. Preclinical studies may be useful for achieving an in-depth understanding of the molecular pathways involved. This, in turn, will favor the possible future treatment of diseases involving a dysregulation of the 5-HT system, such as depression, ADHD, schizophrenia, as well as alcohol and drug addiction (Hauser et al., 2015; Lax et al., 2018).

## Ethics Statement

All experimental procedures have been approved by the ISS animal welfare survey board on behalf of the Italian Ministry of Health (formal license 937/2018-PR, delivered to WA, veterinary surveillance by G. Panzini). Procedures were carried out in close agreement with the directive of the European Community Council (2010/63/EEC) and with the Italian law guidelines. We minimized animals’ suffering and used as few animals as possible, according to the principle of the 3Rs.

## Author Contributions

CC and WA conceived the study. CC and SLR realized the behavioral experiments. EL and ML provided essential tools (the drug). AF and HS realized the affinity radioligand assays. LS provided funding for these experiments. CC with ML and WA wrote a first draft of the manuscript. LS, EA, and HS critically commented on the manuscript. All authors have approved the final version.

## Conflict of Interest Statement

The authors declare that the research was conducted in the absence of any commercial or financial relationships that could be construed as a potential conflict of interest.

## Footnotes

^1^www.newbehavior.com

^2^www.prsitalia.it

^3^www.eurofins.com
